# Advanced and Complex Energy Systems Monitoring and Control: A Review on Available Technologies and Their Application Criteria

**DOI:** 10.3390/s22134929

**Published:** 2022-06-29

**Authors:** Alessandro Massaro, Giuseppe Starace

**Affiliations:** 1 Università LUM “Giuseppe Degennaro”, S.S. 100-km 18, Casamassima, 70010 Bari, Italy; starace@lum.it; 2 LUM Enterprise S.r.l., S.S. 100-km 18, Casamassima, 70010 Bari, Italy

**Keywords:** energy systems, monitoring, energy control strategies, KPIs

## Abstract

Complex energy monitoring and control systems have been widely studied as the related topics include different approaches, advanced sensors, and technologies applied to a strongly varying amount of application fields. This paper is a systematic review of what has been done regarding energy metering system issues about (i) sensors, (ii) the choice of their technology and their characterization depending on the application fields, (iii) advanced measurement approaches and methodologies, and (iv) the setup of energy Key Performance Indicators (KPIs). The paper provides models about KPI estimation, by highlighting design criteria of complex energy networks. The proposed study is carried out to give useful elements to build models and to simulate in detail energy systems for performance prediction purposes. Some examples of energy complex KPIs based on the integration of the Artificial Intelligence (AI) concept and on basic KPIs or variables are provided in order to define innovative formulation criteria depending on the application field. The proposed examples highlight how modeling a complex KPI as a function of basic variables or KPIs is possible, by means of graph models of architectures.

## 1. Introduction

**Figure 1:**
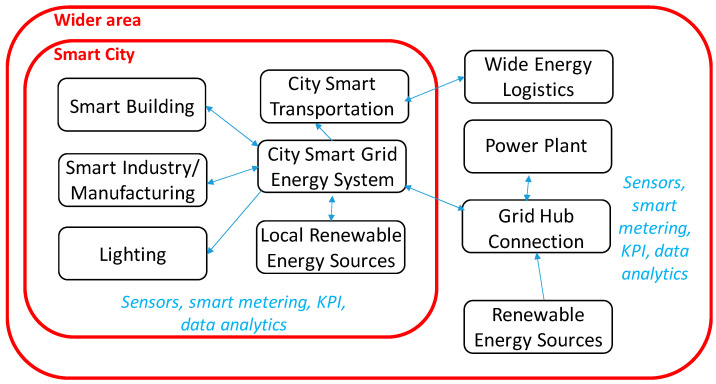
Architecture of an energy monitoring scaled system.

Energy systems correct management includes process modeling, process optimization, hardware and software, appropriate setup design, and monitored operation procedures. Scientific and industrial research often addresses the formulation of new energy strategies. When a high number of variables is to be considered, the energy system modeling becomes complex. To this purpose, scaling the model to propose a framework suitable for simulations and measurements related to the effective energy scenario becomes an important issue. A scaled model representing the main scenario of the proposed research is sketched in Figure 1, where two areas can be distinguished:

(i) a main area related to the control and management of complex logistics fluxes, big power plants, wide grid networks, and renewable energy sources;
(ii) a local area comprising smart cities including smart buildings, local transportation, city lighting, local renewable energy sources, and smart manufacturing energy facilities.

The renewable sources play a very important role in economic and political strategies for energy self-sufficiency of countries. Actually, important technology advances are in the market in terms of biomethane/biogas, photovoltaic, wind, wave, geothermal, hydrogen, thermoelectric, and hydroelectric plants. An important emerging topic for research is the energy harvesting from alternative distributed available sources (light, wind, electromagnetic waves, and vibrations). The full integration of renewable energy sources in complex grid systems takes into account the implementation of sensor and storage systems, and the possibility to apply innovative methodologies for data processing.

Artificial Intelligence (AI) algorithms are increasingly used for data processing, thus providing advanced analytical tools to estimate correlations between variables and predicting different scenarios including energy production, load consumption, and risks. Following the model of Figure 1, an analysis can be carried out about possible innovative hardware and software technologies, to be used for energy measurement and for data processing, by identifying possible Key Performance Indicators (KPIs) modeling and simulating complex energy systems. The KPI estimations are strategic to simulate and to optimize the electrical systems, properly using resources, devices, and loads, addressing the network to low-cost solutions and economic risk-mitigation procedures. Models of complex energy systems are usable to simulate the operation of interconnected hybrid micro-grids and in general grid connections in the small, medium, and large period, supporting the choice of possible combinations of equipments and facilities working in a unique system.

In this scenario, different systems are matched with conventional and renewable energy sources, storage devices, and efficient loads. The output results of the KPIs drive decisions and procedures such as ordinary and extraordinary maintenance services and in-grid/off-grid remote operations, thus ensuring reliable power and decreasing energy costs.

The paper proposes an overview about energy systems by defining possible variables involved in different energy application fields.

### Methodology

**Figure 2:**
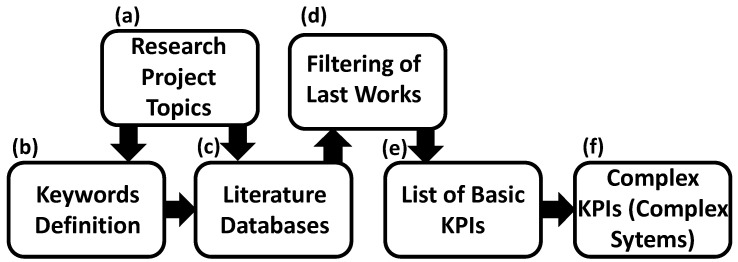
Diagram showing the methodology adopted for this study.

The methodology used in this work is sketched in Figure 2 summarizing the following phases:

(a) following specifications of research projects some topics concerning energy aspects were extracted;
(b) keywords to be used for searching were chosen, such as: *Sensors of Measurements, Smart Energy Meters, Advanced Metering Infrastructures, SCADA, Infrared Thermography, Energy Routing, Energy Technologies, Smart Cities, Renewable Energy, Lighting Control, Power Forecasting, Measurement Approaches and Methodologies, Load Balancing, Load Matching, Solar Radiation Estimation, Microgrids, High Voltage, Data Processing Algorithms, System Grids, Energy KPI indicators, Energy and Manufacturing*, etc.;
(c) searching process over the literature was performed by querying the main international journal databases, especially those focused on energy. The Google Scholar engine was used as well. Open datasets concerning the topics of the examined literature and useful to test AI models were found;
(d) the searching process was optimized on a two-step basis: after a pre-screening, some main works were filtered with a particular interest in the most recent ones; this refinement process allowed us to group the selected papers into four classes: (i) sensors, (ii) technology characterization depending on the application fields, (iii) advanced measurement approaches and methodologies, and (iv) energy KPIs; repetitive older papers were neglected;
(e) the common basic KPIs related the energy aspects were extracted from the selected papers;
(f) criteria were defined to formulate complex KPIs as functions of the basic KPIs or variables.

The complex KPIs are important to model energy systems characterized by a large number of variables. If (*a*, *b*, …) are either significant basic KPIs or measured variables, a complex KPI can be expressed as

KPI = *f*(*a*, *b*, …) (1)

## 2. Sensor Technologies and Energy Metering Systems

Different technologies can be implemented and executed to measure energy parameters. Smart metering technologies [1,2,3,4,5,6,7,8] are suitable for power quality check, measurements of active and reactive power, optimization of grid control, and power consumption monitoring. Supervisory Control And Data Acquisition (SCADA) systems [9,10] are able to integrate measurement systems by controlling parameters in real time. SCADA systems can be used to set up synoptic dashboards monitoring energy and machine/plant parameters; graphical interfaces are typically used to check temperature, electric power, mismatch losses, voltage peaks, and others. Long Range (LoRa) gateway technology and Zigbee protocols are good candidates for horizontal integration of sensors and networks monitoring energy. 

A comfortable technology for measurement of energy efficiency is the infrared thermography [11,12,13,14,15,16] combined with image processing techniques. Sensors can be implemented in complex cloud-connected networks according to the locations of the sites to be controlled. Concerning complex sensor network systems, Zigbee technology [17] and Internet of Things (IoT) devices are appropriate for wireless mesh networks monitoring energy systems. The choice of the network architecture is a function of the data protocol to use and of the data transmission logics. In Table 1, some possible technologies are listed, oriented to sensing and energy metering proposed in the literature and related to the content of this paragraph.

The main issue for the future research on complex systems will likely be combining smart meter measurements using different sensor technologies with communication networks and protocols, so defining architectures suitable to collect synchronized data for KPI evaluation, and to perform real-time control parameters (such as by SCADA systems monitoring through cloud-connected dashboards).

## 3. Application Fields

Energy measurements are required in many application fields both in the civil and industrial sectors. Specifically, applications are in precision agriculture, logistics, buildings, lighting, energy harvesting, wiring, etc. [18,19,20,21,22,23,24,25,26,27,28,29,30,31,32,33].

For what concerns smart buildings, the heating systems are combined with electrical power modules where heating modules could include boilers, cogeneration, heat recovery, and other energy systems to produce heat power. In complex systems, more applications fields are joined, thus increasing the complexity of the model to analyze. In Table 2, the most interesting application fields proposed in the literature are listed.

Other application fields can be found at different scale dimensions with energy being a variable characterizing processes and physical phenomena. The approach to follow to set up KPIs will involve:

- a preliminary study to establish the parameters contributing to the energy behavior of the specific application filed;
- an interaction analysis of elements in the surrounding environment (for example, buildings, cabling, and lighting contributing to the smart city environment).

The KPIs of complex models can be structured in a multilevel architecture where the KPI of a higher level embeds information of all KPIs of lower levels (the root KPI will represent the final indicator of the whole complex system).

## 4. Advanced Measurement Approaches and Methodologies

Measurement approaches and methodologies [34,35,36,37,38,39,40,41,42,43,44,45,46,47,48,49,50,51], such as sensor allocations and related protocols, mainly involve data processing techniques. Different data analysis tools can be applied to extract more information, optimizing energy systems such as predictions, parameter classifications, and possible unbalanced energy conditions. Supervised and unsupervised AI algorithms represent advanced solutions extracting hidden information and realizing Decision Support Systems (DSSs) for energy management. In Table 3 some methodologies proposed in the literature are listed.

Combining different approaches (for both measuring and processing data) to extract more and new information useful for the definition of new efficient KPIs will be the key concern for researchers in the future.

## 5. Energy KPI Indicators

KPIs are fundamental to estimate the energy efficiency of a system and are specific for the application to be considered [52,53,54,55,56,57,58,59,60,61,62,63,64,65,66,67,68,69]. Complex KPIs can be formulated as a combination of more KPIs properly taking into account weights for each parameter. The weights of the variables to assign come from the related importance of the specific KPI. KPIs can refer to energy efficiency, energy quality, economical and business aspects, losses, pollution, consumption, and sustainability. In Table 4, KPIs for energy systems are reported and commented upon.

The proposed state of the art is quite exhaustive about standard indicators including costs, losses, quality, and pollution. Complex systems, such as sustainable energy systems in a large scale (green economy), could require the use of more of these KPIs which can be furthermore interrelated.

## 6. Discussion: Research Topics Correlated to Energy Complex Models 

Basic KPIs and variables discussed in Table 1, Table 2, Table 3 and Table 4 can be associated with each element (subsystem) of the energy system of Figure 1. In Table 5, the references matching the ten subsystems are grouped.

KPIs of complex energy systems are estimated by processing a big quantity of variables. Distributed energy systems require a high computational cost for data processing. In this trend, quantum computing and related frameworks could support this weakness [70]. For the energy applications, another main issue correlated to the data extraction for processing is the communications systems choice which requires optimized networks [71,72]. Quantum computing represents a powerful solution for complex systems data processing when applications deal with fossil, renewable, or nuclear energy, even when different aspects such as energy management, efficiency of innovative materials, grid security, and simulations [73] have to be addressed. Quantum algorithms and quantum computing approaches are also suitable for electrical grid operation planning [74,75]. Energy cloud management [76] and big data analytics [77] become fundamental tools when upgrading to energy data processing issues, especially concerning electricity load forecasting where large datasets are required for modeling self-learning of the AI supervised algorithms.

**Figure 3:**
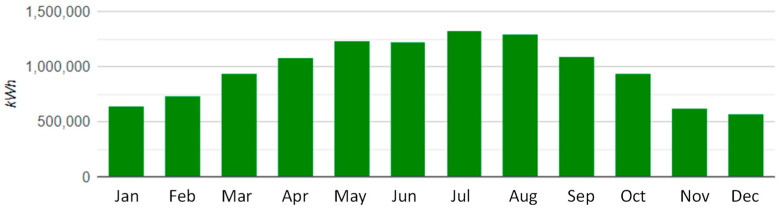
Yearly production for an 8 MW photovoltaic plant with 20,000 30 deg slanted panels of 400 W_peak_ each. The calculus was performed for an installation in Lecce (southern Italy). Yearly PV production: 11,710,400 kWh.

Pollution monitoring is a research topic too, as carbon dioxide, carbon monoxide, unburned hydrocarbons, particulate matter, sulfur dioxide, and nitrogen oxides emissions have to be counted to match ever higher environmental prescriptions [78,79,80]. To this goal, estimating parameters strictly correlated to the green sustainability indexes becomes of high importance, such as Carbon Footprint (CF) (a parameter taking into account greenhouse gas emissions towards the atmosphere caused, for example, by an energy system in the construction of components, during the operation, and when dismissed). For example, the release of about 6,218,222.4 kg_CO2_/year (see Figure 3) can be avoided by installing an 8 MW photovoltaic plant to match the electrical energy needs in the south of Italy. The CF estimation is possible by considering the “factor of emission of the electricity mix” which represents the average value of CO_2_ emissions due to the production of electricity in Italy. The factor is provided in Italy by the Ministry of the Environment and is 0.531 kg_CO2_/(kWh year).

Concerning renewable energy, data of environmental pollution due to energy generation can be analyzed by means of different tools such as drones (such as for water quality in solar farms by applying underwater image detection [81]), acoustic signal processing in biodiesel production [82], the Life Cycle Impact Assessment (LCIA) approach determining resource consumption and substance release in the environment [83], and a multivariate time series method predicting air pollution [84]. 

Further important research topics concern the energy storage technologies [85,86], and the related operational approaches [87,88,89,90]. The impact of deep refurbishment and the use of renewable energy sources of buildings can be significant when passing from a single building level to a district scale [91]. In addition, the energy model can be more complex when a capillary distribution in the city is considered for small energy producers such as owners of small wind turbines [92], and hybrid solutions combining solar radiation, wind power, and biomass [93]. Numerical tools and data mining platforms such as Konstanz Information Miner (KNIME) [94,95,96] can support the calculus of complex structured indicators by applying AI data processing. In Appendix A, an example of KNIME data processing predicting PV power is reported. 

The monitoring of energy consumption in industrial applications can be optimized by the energy manager who manages data processing and processes correlated to the primary energy consumption [97]. A complex energy system takes into consideration many processes related to energy consumption and production as can happen in industrial applications. In this scenario, process mining implementation (processes automatized by AI controls [98]) could support process implantation and data-driven efficient energy strategies. 

Energy Management Systems (EMSs) [99,100] represent important applications and research topics. Different rule-based strategy models are proposed in the literature. Some authors discuss control approach schemes with an operation process for micro-grid systems including forecasting, sensing, and actuation [99]. The energy management problem is typically formulated as a deterministic Optimal Control Problem (OCP) [100]. 

Other EMS approaches are mainly focused on the analysis of management uncertainties such as fuzzy-based methods, linearization approach, probabilistic method, Monte Carlo method, Gaussian mixture model, estimation distribution and stochastic models [100]. Probabilistic methods are classified as numerical and analytical ones [100]. Hybrid approaches are possible such as scenario based and probabilistic approaches [100]. Control and optimization processes play an important role in EMSs [101].

AI algorithms are proposed as real-time application optimization control algorithms for energy management strategies for hybrid power engines [102], thus suggesting a similar use for a general energy system equipped with an AI supporting decision management. Concerning electricity market bidding, some authors analyze a theoretical framework of energy management optimization, by taking into account the interaction between the Independent System Operator (ISO) agent, commercial user agent, and power plant agent [103].

## 7. Conclusions and Perspectives

The paper focused on an overview of technologies, KPI investigation and definition, measurement approaches, and data processing methods, spread out over different energy application fields covering civil and industrial scenarios. The specific literature analysis defines many aspects which have to be considered when more complex systems characterized by multilevel KPIs processing different input parameters are addressed. The present review highlights important elements to be considered in real applications modeling advanced energy systems that manage a large number of variables, including the AI concept improving KPIs or defining new ones.

Complex KPIs can be modeled by architecture based on nodes linked into a unique graph. Each node can represent a Basic Variable (BV), a Basic KPI (BK) over a Complex KPI (CK) formulated as a combination of a BV and BK as in Equation (1). The nodes representing the CK behave as a “supernode” [104]. Each node belongs to a hierarchical level. Different levels represent the whole complex system. The KPI design criteria based on a hierarchical or a multilevel approach allow one to better distinguish the energy efficiency of a single element of the whole energy system. The KPIs characterized by a higher level will contain the information of lower levels. The lower KPIs or variables will be independent from KPIs of higher levels. 

A main application field is that of the smart buildings, where energy control and management involve a large number of electric loads and plants, especially if large indoor areas are considered. The formulation of complex KPI systems can define correlated indicators supporting the full energy management process, which can be performed by:

- a cloud framework;
- reading signals detected by sensors;
- processing data by means of AI algorithms predicting daily loads, optimizing energy consumption and loads;
- switching electric power (as for energy routing applications).

KPIs in smart buildings could take into account other important aspects such as wellness/security (gas sensing) and can be matched with home automation applications. The KPI model can be more complex if more buildings are considered in the same system to be analyzed; all the KPIs of all the buildings can be combined to define a unique one for a neighborhood or a whole city. The modularity of the model is then useful to scale the application for wider areas. An example of complex KPIs in smart building is provided in Appendix B.

Energy KPI models can also be formulated in particular application fields such as logistics. Actually, logistics applications are commonly characterized by energy aspects. A logistics system can be characterized by different variables contributing directly (vehicle load factor, cargo weight, router length, specific fuel consumption, vehicle kerb weight, etc.) or indirectly (such as for the driver behavior which can influence the vehicle consumption). The KPI model will be useful to optimize logistics fluxes based on the energy behavior model of the fleets. More complex systems can be associated with the joined actions of different vehicles involved in the transportation of the same product (transport by truck, plane, train, ship, etc.), and different logistics networks composed of different hubs. An example of complex KPIs in logistics is provided in Appendix C.

Concerning renewable energy systems, the KPI model can be characterized by different elements such as renewable sources, local electrical networks (medium-voltage electrical cabling and electrical components of the site where the energy sources are allocated), and high-voltage networks. The complexity of the system is increased when different renewable energy fields are considered; the monitoring and control of more PV fields (structured in subfields) transmitting energy to a high-voltage power plant is an example of a complex system. An example of complex KPIs in photovoltaic plants is provided in Appendix D.

## Figures and Tables

**Table 1:** State of the art: sensor technologies and energy metering systems.

Technologies/Metering Systems	Topic	Description	Ref.	Basic KPI or Variable
Smart Metering	Unbundled Smart Meters (USMs) and Next-Generation Open Real-Time Smart Meters (NORMs)	Grid-tied inverter control	[1]	Voltage, current, instantaneous power, fault signal trend
	Power Quality (PQ) meters	Measurements of active/reactive energy, active/reactive power, frequency, Root Mean Square (RMS) voltage/current, FFT, Total Harmonic Distortion (THD)	[2,3,4]	Voltage in percentage [2]; annual active energy heat view, and nonlinear load analysis [3]; sampling data granularity [4]
	Simultaneous Wireless Information and Power Transfer (SWIPT) technique	Energy efficiency optimization considering Orthogonal Frequency Division Multiplexing Distributed Antenna System (OFDM-DAS) with Power Splitting (PS)-SWIPT system	[5]	Power harvester, energy harvester, energy, spectral efficiency [bits/s/hertz], energy efficiency [bits/Joules/hertz]
	Long Range (LoRa) gateway technology	LoRa protocol network for communication between the smart meters and the gateway	[6,7]	Data granularity, current, voltage and power weekly distributions, Wi-Fi coverage, and packet loss rate (PLR) [6]; power load, load voltage, humidity, and temperature [7]
	Advanced Metering Infrastructure (AMI) with data aggregation points	Metering system collecting power consumption data from all smart electrical appliances and adopting unsupervised clustering algorithms	[8]	Signal-to- Interference-plus-Ratio (SINR)
SCADA	Energy Management System (EMS) developed using distribution Supervisory Control And Data Acquisition (SCADA)	System controlling devices used in Heating, Ventilation, and Air Conditioning (HVAC) and lighting systems across multiple locations	[9]	Fault occurrence, addition of loads, phase balancing
	Data acquisition and remote monitoring systems for micro-grid	Data acquisition solar–wind–biogas integrated micro-grid system (Raspberry Pi technology)	[10]	Smart meters of Elite 440–443 series of Secure Pvt. Ltd.PN: voltage, PP voltage, power factor, active power, apparent power, active/apparent forwarded energy, reactive lag/lead forwarded energy, phase angle, THD voltage, THD current, THD power
Infrared Thermography	Control the temperature of the overhead conductor	Estimation of the temperature of the power lines	[11]	Infrared thermometer temperature [°C], Pt100 temperature [°C], solar radiation [W/m^2^], current [A], ambient temperature [°C], relative humidity [%], perpendicular wind speed [m/s]
	Photovoltaic panel checking defects	Application of the clustering and of thermal pixel counting algorithms to the radiometric image enhancing panel defects	[12,13]	Infrared radiometric temperature [°C], total energy produced and predicted by ANN [kWh] [12]; infrared radiometric temperature [°C], % of PV panel variation versus temperature [13]
	Radiometric image processing of thermal insulation PVC composite panels	Evaluation of thermal losses of building panels along the aluminum junctions	[14]	Infrared radiometric temperature [°C], homogeneity of aluminum panel junctions (PV)
	Application in energy router system	Applications for monitoring of loads, energy source devices, and energy storage systems	[15]	Infrared thermometer temperature, load prediction, weather forecasting, calculation of energy needs
	Thermal dispersion evaluation in indoor environments	Data mining (k-means algorithm for clustering and the Nearest Neighbor (NN) for classification) enhancing thermal dispersions	[16]	External temperature, room temperature, classification of parts of thermal image (image processing evaluating the risk of the heat leakage)
Zigbee	Wireless technology able to exchange motion data of human movement in rooms with a centralized air conditioning unit	Switching off of centralized air conditioning unit (reducing unused electricity)	[17]	Display when an area served by an AHU unit is without users, number of empty rooms versus days

**Table 2:** State of the art: application fields of energy metering.

Application Field	Topic	Description	Ref.	Basic KPI or Variable
Precision agriculture	Precision agriculture reducing the use of resources (energy, water)	Internet of Things-based systems for greenhouse sensing and actuation	[18,19]	Temperature, light detection by a photo resistor (measurements in a greenhouse) [18]; monitoring energy consumption and control of photovoltaic generation (to enable powering devices only when needed) [19]
Logistics	Logistics KPIs based on energy aspects	Indicators based on fuel consumption, vehicle kerb, weight, engine stress, maintenance level	[20,21,22]	Load factor, cargo weight, router length, specific fuel consumption (liters consumed every 100 km), vehicle kerb weight [20,21]; energy and fuel consumption (driver costs) [22]
Buildings	Building Energy Management System (BEMS)	Heating, Ventilation, and Air Conditioning (HVAC) system reducing energy consumption	[23]	Temperature, humidity, and ambient lighting
	Smart building architecture with IoT sensing devices and communication network protocols	Energy consumption monitoring, uploading data to a cloud server	[24]	RMS, Fourier series, Power Factor (PF), active power, reactive power, energy, Total Harmonic Distortion (THD)
	Building energy management system and home automation	Temperature and illuminance wireless sensor nodes with energy harvesting and Zigbee modules	[25]	Temperature and illuminance
Lighting	Smart public lighting control and measurement system	Smart cities monitoring streetlights by LoRa network	[26]	Horizontal illuminance E [lux], KPI about the illumination level has a function in relation to time and pedestrian flow (total energy saved, regulation percentage, %Reg)
	Energy Management System (EMS) by Internet of Things (IOT) for lighting control	IoT technology for lighting control for a university campus, providing energy savings by eliminating standby consumptions and adapting the user behavior to the real environmental conditions (building map construction)	[27]	Human occupancy patterns
	Public lighting control	Energy saving technologies turning on/off streetlights automatically	[28]	Distance detection switching on the light when the object is sensed in a nearby area
Energy harvesting measurement system	Wave Energy Converter (WEC)	Floating buoy with sensors collecting data processed by machine learning algorithms	[29]	Output power of wave energy harvester system
	Energy harvesting system from water flow	IoT-based energy monitoring system monitoring the amount of harvested energy	[30]	Output voltage [mV] versus distance between sensor and water source [cm], output voltage [mV] versus number of piezo sensors, output voltage versus water flow rate expressed in liters per second, output voltage [mV] versus temperature [°C], output voltage [mV] versus angle between water flow direction and sensors [Degree]
	Road vibration energy harvesting	Vehicle move sensor generating electrical energy by using the pressure of the vehicle’s weight	[31]	Voltage
Electrical cable connection check	Multisensor monitoring system for medium voltage cable electrical joints	Sensor node including radio, sensors, and energy harvester checking degrading cable connections for medium-voltage grids	[32]	Current, Partial Discharge (PD), fault current, over-temperature, vibration (measuring external shocks)
Energy production monitoring in industry	Energy consumption monitoring in production	Multisensor system based on the reading of electrical power consumption of different production machines	[33]	Power of production machines

**Table 3:** State of the art: advanced measurement approaches and methodologies.

Measurement Approaches and Methodologies	Topic	Description	Ref.	Basic KPI or Variable
Bayesian model	Energy measurements	Energy measurement and verification by Bayesian model; International Performance Measurement and Verification Protocol (IPMVP) solution by Bayesian approach	[34]	Energy [kWh] versus cooling degree days
Load forecasting	Load forecasting Weighted Least Square (WLS) state estimation algorithm for micro-grids and network splitting problems	Load information obtained by forecasted, historical data, and by smart real-time meters; monitoring of switching devices	[35]	Active power, reactive power, loading %, Power Factor (PF), voltage magnitude error, voltage angle error, bus voltage magnitude uncertainty %, versus bus number, deviation between the simulation results regarding the estimated status of the switching devices and their true status
	Cloud electric load switching in buildings, and electrical outlet management predicting exceeding thresholds	Long Short-Term Memory (LSTM) neural network algorithms able to control, to activate, and to disable electrical loads connected to multiple outlets placed in a building and having defined priorities	[36]	Current, total electrical current of outlets, global active power
Power forecasting	Adaptive Solar Power Forecasting (ASPF) method for precise solar power forecasting	Combination of data clustering (k-means), variable selection, and neural network optimizing solar power forecasting	[37]	Output power [kW] versus time [h], sunshine duration, relative humidity, air temperature
	Power load prediction for rural electrical micro-grids	Long Short-Term Memory (LSTM) Artificial Neural Network (ANN) algorithms	[38]	Output power versus time, power load prediction, measured power load versus predicted power load
Data analysis	Error minimization by mathematical model for smart metering system optimization	Identification and minimizing the measurement errors to optimize the electricity readings’ accuracy and to reduce the electricity losses and related costs	[39]	Own Technological Consumption (OTC) as the difference between the energy entered in the commercial contour and the energy distributed to the consumers versus time (months of the year)
	Data-driven approach for large distribution grids	Decentralized Pruned Physics-Aware Neural Network (D-P2N2) estimating power losses	[40]	Estimated voltage magnitude in different scenarios of node distribution
	Network loss energy measurement based on machine learning	Machine learning algorithm calculating network loss to obtain the optimal load distribution map	[41]	Prediction of network losses and loads
	Solar radiation estimation and forecasting by ANN	Models estimating solar data at a specific time to optimize management of energy and to anticipate the production/consumption balance	[42]	Estimated Global Horizontal Irradiation (GHI) [Wh/m^2^] versus measured GHI [Wh/m^2^], 5-min solar irradiation [Wh/m^2^] versus time [h], global solar irradiance [W/m^2^] versus time [h], direct normal irradiance [W/m^2^] versus time [h]
	Decision Support System (DSS) to classify and optimize the energy efficiency	Prediction of energy efficiency by Zigbee sensors placed in strategic locations in a smart building	[43]	Mean compressor active power versus date
Energy routing	ANN-based reinforcement learning method optimizing energy routing design	Energy Internet (EI) model and ANN algorithm managing the optimal energy routing path	[44]	Electrical demand [kW] versus time [h], thermal demand [kW] versus time [h], PV output power [kW] versus time [h], voltage of ports connected with connection lines [kV] versus time [h], electrical power [kW] versus time [h]
	Software-Defined Networks (SDNs) enabling 5G monitoring systems	Technique exploiting the network combined with traffic engineering techniques in order to reduce the overall power consumption and the number of active links	[45]	Average energy savings [%] versus number of network controllers, average number of pruned links [%] versus number of network controllers, cumulative distribution function of link utilization varying the amount of controllers in different areas
Wind speed forecasting	LSTM predicting wind speed	LSTM-based models improving the forecasting accuracy	[46]	Maximal Information Coefficient (MIC) measuring the predictability of wind speed series versus delay time [min], wind speed components [m/s] versus time [min], forecasting error [m/s] versus number of forecasting samples
Selection of metering points	Optimal location of metering points in grid distribution for power quality metering and assessment	Approaches to use for complex energy distribution systems	[47]	Cost function associated with metering point allocation
Networked wireless control systems	Wireless Sensor Network (WSN)	New communication protocol for energy efficiency and evaluation of the network global energy consumption levels	[48]	Energy consumed by a network responsible for the transport of the control signal
Energy measurement	Energy measurement approach in high-voltage power networks at low currents	Approach for measuring system operating out of precision specification	[49]	Low current
Energy flow management systems	Energy model applied for residential premises	Statistical methods for the assessment of the energy model using as input data measured temperature	[50]	Temperature
	Cyber-enabled grids (energy management)	Cloud sensing and actuation for physical world (power grids)	[51]	Current, voltage, and measurement approaches

**Table 4:** State of the art: energy KPIs.

Indicator	Application Field	Description	Ref.	KPI Classification
Energy efficiency in industries	Energy efficiency indicator by utilizing data collected from the textile industry in EU member states	TFEE indicator (ratio of target energy input to the actual energy input) by also taking into account policy goals of energy saving, pollution reduction, and sustainable economics	[52]	Energy efficiency
	Industrial needs	Energy management in production and role of KPIs	[53]	Energy management efficiency
	Energy-based KPIs	Exergy-based performance indicators in industry (total exergy efficiency, task exergy efficiency, exergy efficiency disregarding transiting exergy, specific exergy-based indicators, environmental exergy-based indicators)	[54]	Energy efficiency
	Energy efficiency indicator in manufacturing sector	Measurement efficiency of the energy efficiency of manufacturing activities from factory level to process and product level: ○ energy costs by type/kiloliters produced; ○ energy consumption/kiloliters produced; ○ energy consumption directly taken from monthly invoices; ○ (electricity produced by trigen. + PV)/(sum of electricity produced on-site + electricity purchased); ○ (electricity produced + HRSG * output + absorption chiller)/(generators gas consumption); ○ 1 − ((sum of energy purchased in current month)/(sum of energy purchased in corresponding month of previous year))	[55]	Economic energy efficiency
Energy efficiency of components	Wind turbine energy efficiency index	SCADA monitoring parameters of wind turbine such as loss of heat and temperature, key performance indicators for operational management of wind turbines estimating KPI (power, wind conditions, wind speed, full load hours, energy consumption, data availability, site quality, operating hours, etc.)	[56,57]	Energy monitoring efficiency
	Energy efficiency indicators for water pumping systems in multifamily buildings	Design guidelines for water pumping systems to serve vertical multifamily buildings	[58]	Energy system design optimization
Energy quality	Energy quality control for the power supply systems of electrical devices and systems	Harmonic composition monitoring system by fluxgate sensors (noninvasive monitoring)	[59]	Energy quality
	Power Quality (PQ)	Statistical Signal Processing (SSP) and intelligent methods for PQ analysis, PQ and reliability characterization, management of PQ big data for smart grid, PQ monitoring systems (architectures and communications), PQ losses and mitigation assessment, new PQ monitoring norms and standards	[60,61,62,63,64,65]	Energy quality
Energy KPIs	Sustainability in urban areas	Electrical performance KPIs (Electrical Self-Production (ESP), Electrical Self-Production from Renewable Energy Sources (ESPRES), Electrical Self-Production from Combined Heat and Power (ESPCHP)); Thermal performance KPIs (thermal energy produced by means of electric boilers (TB), thermal energy produced with combined heat and power (TCHP), thermal energy produced by means of heat pumps (THP), thermal energy produced by renewable energy sources (TRES), Global Self-Production from CHP (GSPCHP)); Environmental impact KPIs (tons per year of avoided CO_2_^−^ECO_2_^−^, NO_x_^−^ENO_x_^−^, and SO_2_^−^ESO_2_^−^)	[66]	Energy sustainability
	Renewable Energy Source (RES) KPIs	% share of RES for electricity, heating/cooling, and Domestic Hot Water (DHW), % share of Decentralized/Distributed Energy Resources (DERs), % reduction of the power peaks, generation forecasting accuracy, energy losses, % voltage variations, on-site energy ratio, Maximum Hourly Surplus–Deficit (MHS-Dx), Reduced Energy Curtailment of RES/DES, grid congestion, battery degradation rate, System Average Interruption Frequency Index (SAIFI), System Average Interruption Duration Index (SAIDI), unbalance of the three-phase voltage system, harmonic distortion, storage energy losses, degree of PV self-supply, frequency control, Energy Return on (Energy) Investment (EROI), CO_2_ tons saved, % noise pollution exposure, reduced fossil fuel consumption (TOE/year), carbon footprint of heating houses (Kg CO_2_/year), economic KPIs, social KPIs, legal KPIs	[67]	Energy efficiency
	Building-level energy performance indicators	Total energy use, life cycle building energy use, Electrical Load Factor (ELF), Energy Use Intensity (EUI), Energy Performance Coefficient (EPC), building efficiency index, EnergyStar Score, Zero Energy Performance Index (ZEPI), Home Energy Rating System Index (HERS), Smart Readiness Indicators (SRIs), whole building performance indicator, Lighting Power Density (LPD), Daylight Effectiveness Indicators (DEIs), Total System Performance Ratio (TSPR), HVAC operational consistency indicator, Load Energy Ratio (LER), HVAC Energy Efficiency (η(HVAC)), plug-load off-hours ratio, Coefficient of Performance (COP), Energy Efficiency Ratio (EER), Seasonal Energy Efficiency Ratio (SEER), Heating Seasonal Performance Factor (HSPF), Integrated Part Load Value (IPLV), boiler efficiency η, luminous efficacy, Fan Energy Index (FEI)	[68]	Energy efficiency
	Flexible buildings and reliability of the electric power	Load cover factor, supply cover factor, Loss of Load Probability (LOLP), energy autonomy (1-LOLP), mismatch compensation factor, On-site Energy Ratio (OER), Grid Interaction Index (GII), no grid interaction probability, Capacity Factor (CF), connection capacity credit, One Percent Peak (OPP), Peaks Above Limits (PALs), absolute grid support coefficient, relative grid support coefficient, equivalent hours of storage, Flexibility Factor (FF), Flexibility Index (FI), procurements cost avoided flexibility factor, volume shifted flexibility factor, available structure storage capacity, storage efficiency, available electrical energy flexibility efficiency, flexible energy efficiency	[69]	Energy flexibility

**Table 5:** References including basic KPIs and energy variables associated with the subsystems of Figure 1.

Sub System	References Mainly Indicated for Basic KPIs or Variables and Associated Research Topics	Main Key Energy Variables
(A) Smart Building	[9,16,17,23,24,25,26,27,34,35,36,43,49,50,66,68,69]	Lighting power electricity, temperature, load power electricity
(B) Smart Industry/Manufacturing	[6,7,9,17,23,24,33,53,54,55,66,68]	Machine power electricity, temperature (energy losses)
(C) Lighting	[25,26,27,28,68]	Illuminance, lighting power density
(D) City Smart Transportation	[20,21,22,26,31]	Fuel consumption
(E) City Smart Grid Energy System	[1,2,3,4,5,6,7,8,9,10,12,13,14,16,17,28,31,34,35,36,37,38,39,43,44,45,48,49,50,66,68,69]	Current, electrical power, power distributed in the grid, electrical losses
(F) Local Renewable Energy Source	[1,2,3,4,5,6,7,8,12,13,14,15,29,30,31,37,42,46,59,60,61,62,63,64,65,66,67]	Power generated
(G) Wide Energy Logistics	[20,21,22,26,31]	Fuel consumption
(H) Grid Hub Connection	[1,2,3,4,5,6,7,8,11,32,39,40,41,44,45,47,48,50,51]	Electrical power losses (energy efficiency)
(I) Renewable Energy Source (wider areas)	[1,2,3,4,5,6,7,8,10,12,13,15,18,19,29,30,32,34,35,36,37,41,42,46,51,59,60,61,62,63,64,65,67]	Electrical power generated

**Figure A1:**
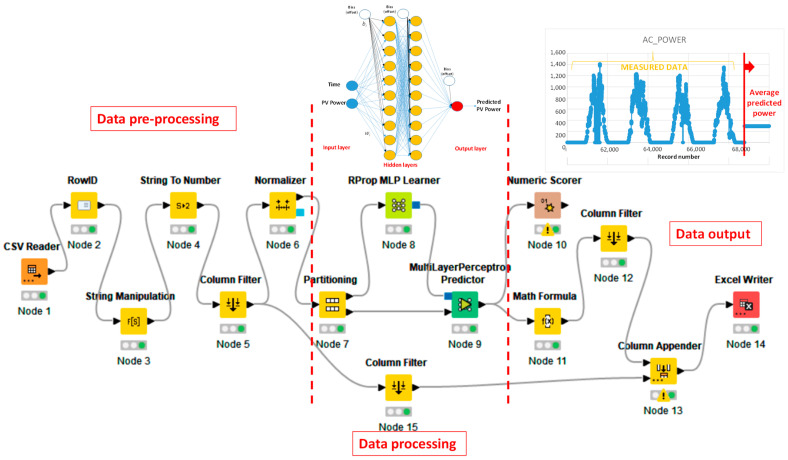
KNIME workflow implementing ANN-MLP algorithm to predict AC power generated by a PV plant. Insets: equivalent ANN-MLP network and predicted average AC power (expressed in kW) processing the dataset [105].

**Figure A2:**
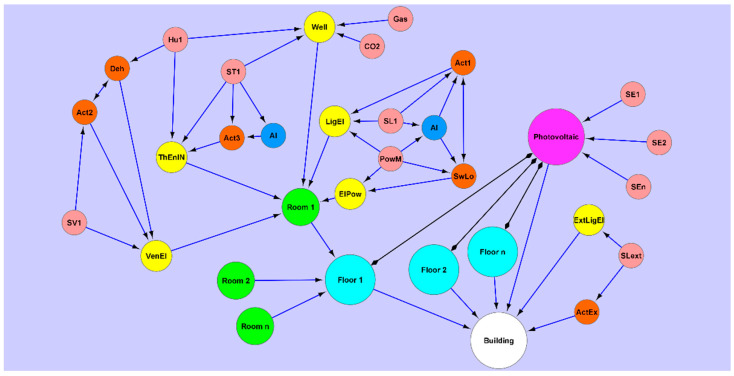
Complex architecture in smart building energy system. The graph model with linked nodes has been designed by means the open source *Cytoscope* tool.

**Figure A3:**
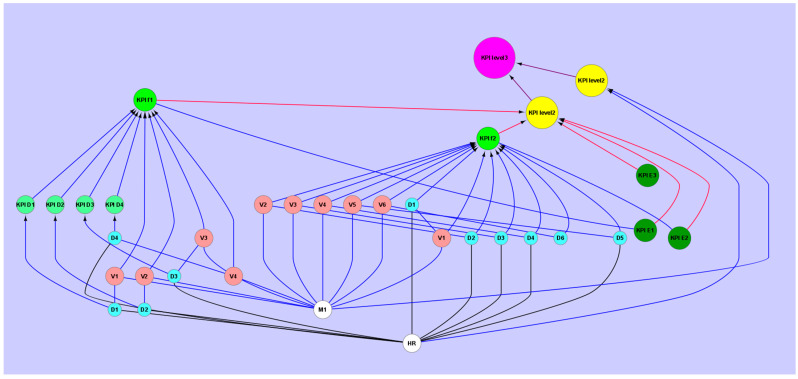
Complex architecture in smart logistics transport systems. The graph model with linked nodes was designed by *Cytoscope*.

**Figure A4:**
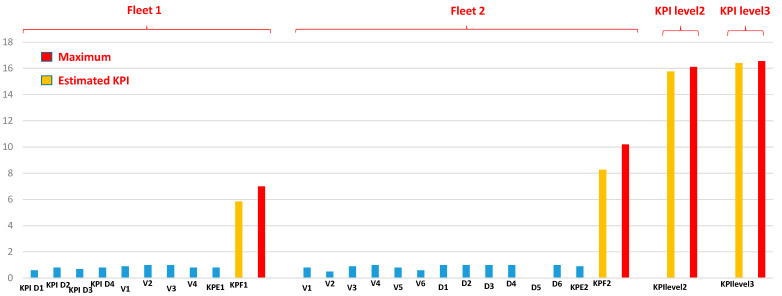
Example of KPI simulation related the model of Figure A3 using Equation (A1) for the calculated KPIs.

**Figure A5:**
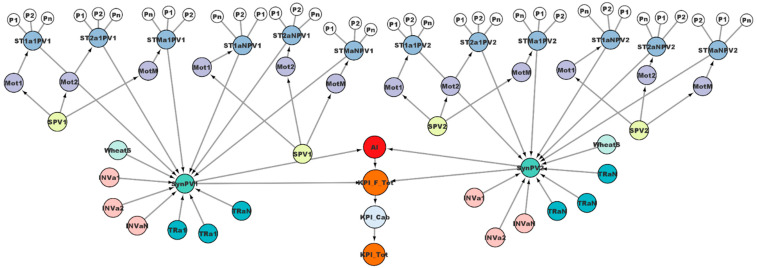
Complex architecture controlling two PV fields and related high-voltage network. The graph with linked nodes has been designed by *Cytoscope*.

## Appendix A

KNIME is an open source tool suitable for AI data processing. Different supervised and unsupervised data mining tools are available as libraries implemented in objects (nodes) linked as a workflow. Figure A1 illustrates an example of KNIME data processing testing the dataset found in [105] (solar power generation and sensor data of PV power plants). The open dataset is typically used to test data models supporting the choice of the algorithm to adopt for forecasting or for classification. The workflow is structured in three main parts:

- data pre-processing, preparing the dataset to process (filtering, data cleaning, normalization, etc.);
- data processing (data processed by AI algorithms);
- data output (results, algorithm scoring, data storage).

In the proposed example (Figure 3), an Artificial Neural Network Multilayer Perceptron (ANN-MLP) supervised algorithm is executed (identification of the electrical power as labeled class to predict), processing a dataset of 68,777 records. Other open datasets useful to test AI algorithms, and matching the topics of the paper, can be found in the Kaggle database, and are related to wind turbine power [106,107,108,109,110], solar power generation [111,112,113,114], smart buildings [115,116], energy generation and consumption [117,118,119,120], smart grid stability [121,122], fault detection [123,124], and energy demand [125].

## Appendix B. Example of Complex KPI in Smart Building

Energy control and management in smart building applications can involve different variables according to loads and plants, especially if large indoor areas are considered. The full energy management process can be performed in a cloud framework, by reading signals detected by sensors, and processing data by means of AI algorithms predicting daily loads, optimizing energy consumption and load and switching electric power (as for energy routing applications). In Figure A2, an example of a complex system integrating data sensors in smart building is shown; the system is able to detect basic energy parameters and to enable loads as a function of data processing. In the proposed example, an efficiency indicator characterizes each floor. In the model, indicators are included estimating photovoltaic and thermal/electrical efficiencies by means of KPIs and different sensor data. All the KPIs of each floor are combined into a high-level KPI representing the energy efficiency of the whole system. The system of Figure A2 takes into account both sensing and actuation functions including AI data processing. All the AI algorithms can be executed into a unique platform as an AI engine. The terms used in the architecture and the meaning of each term are discussed below (BV is a basic value, BK is a basic KPI, CK is a complex KPI as a function of BV and BK).

- Room i = *f*(ThEnIN, ElPow, Well, LigEl, VenEl): total room efficiency KPI (CK);
- Floor i = *f*(Room 1, Room 2,…Room n, Photovoltaic): total floor efficiency KPI (CK);
- Building = *f*(Floor 1, Floor 2,…Floor n, ExLigEl): total building efficiency KPI (CK);
- VenEl = *f*(Act2, Deh, SV1): ventilation electricity indicator indicating power consumption KPI (as a function frequency of Act2 and Deh activations, and on SV1) (CK);
- ThEnIN = *f*(Act3, ST1): thermal energy indicator (KPI as a function frequency of Act3 activations, and on ST1) (CK);
- SV1 = *f*(air flux/velocity): sensor of ventilation measuring air flow from window (BV);
- Act1 = *f*(SL1, AI): actuator of lighting (actuation based on AI prediction in the short period and on SL1);
- Act2 = *f*(Deh, SV1): actuator for ventilation synchronized with Deh (actuation based on SV1);
- Act3 = *f*(ST1, AI): water heater actuator for heating (actuation based on AI prediction of building external temperature and on ST1);
- Deh = *f*(Act2, UM1): dehumidifier actuator synchronized with Act2 (actuation based on UM1);
- AI = *f*(SL1, PowM, ST1): artificial intelligence predictor algorithm;
- Hu1: humidity sensor measuring relative percentage humidity (BV);
- ST1: sensor of temperature measuring indoor temperature (BV);
- Well = *f*(Hu1, ST1, CO_2_, Gas): wellness indicator (CK);
- Gas: gas sensor monitoring air pollution coming from automobiles (external pollution) such as nitrogen oxides, NOx (NO and NO_2_), and carbon monoxide (CO) (BV);
- CO_2_: carbon dioxide sensor (BV);
- SL1: sensor of lighting measuring illuminance and enabling Act1 (BV);
- LigEl = *f*(Act1, SL1, PowM): lighting electricity KPI (CK);
- ElPow = *f*(PowM, SwLo): electrical power indicator including switching load efficiency (CK);
- PowM: power meter (BV);
- SwLo = *f*(AI, Act1, PowM): energy router actuator managing switching load;
- Photovoltaic = *f*(SE1, SE2,… SEn): photovoltaic synoptic monitoring solar radiation and PV (hardware and software units/modules);
- SEi: solar radiation sensor measuring solar energy (BV);
- ExLigEl = *f*(SLext): external lighting electricity indicator (CK);
- SLext (BV): sensor of external lighting measuring power consumption of external lights and external solar illuminance;
- ActEx: actuator for external lighting.

The complex model of Figure A2 takes into account other important aspects such as wellness/security (gas sensing) and can be matched with home automation applications. The model can be more complex if other buildings are considered in the system to be analyzed. In this case, combining the KPIs of all the buildings, defining a unique one concerning a neighborhood or a city, is also possible. The modularity of the model is then useful to scale up the application to wider areas. The complex KPI referring to the building can be modeled by Equation (1).

## Appendix C. Example of Complex KPI in Logistics

Logistics applications are deeply characterized by energy aspects. A logistics system can be characterized by different variables contributing directly or indirectly (such as for the driver behavior which can influence the vehicle consumption) to the estimation of the KPIs. In Figure A3, an example of a complex system associated with estimated KPIs in logistics is illustrated.

Specifically, the system of Figure A3 defines a model estimating KPIs in three main hierarchical levels, where the final complex KPI (KPI level 3) is the total KPI (“Energy” KPI) including the calculation of all the parameters contained in the analyzed model. In the example of Figure A3, the KPIs refer to two hypothesized vehicle fleets traveling through two different country regions, and each KPI can be expressed, similar to Equation (1), by the following linear function:

KPI *=* ay_1_ + by_2_ + … (A1)

where a, b,… are the weight coefficients and y_1_, y_2_, … are the parameters/variables defined in Table A1. Each KPI must be properly normalized in order to use consistent scales.

**Table A1:** KPIs in “Energy logistics” *(BV means basic values, BK means basic KPI, CK means complex KPI as a function of BV and BK)*.

Level	KPI	Description
1(CK)	$KPI_{D_{i}}=f\left(D_{s},D_{e},\ldots \right)$	$KPI_{D_{i}}$ that is the KPI of the driver $D_{i}$ ($D_{s}$ (BV) is the parameter estimating the effect of the average velocity provided by a GPS, the revolutions per minute (rpm) (BV) accelerations, and other engine parameters (data provided by the engine control unit); the parameter $D_{e}$ (BV) represents the driver efficiency correlated to a correct driving style (use of AI algorithm).
1(CK)	$\begin{array}{c} KPI_{V_{i}}=V_{i}=\\ =f\left(\begin{array}{c} \gamma ,CW,RL,sfc,vkw,\\ ES,fc,ml \end{array}\right) \end{array}$	γ (BV): vehicle load factor (filling factor of the space dedicated to the product loading); CW: cargo weight (BV); RL: router length (BV); sfc: specific fuel consumption as L/100km (BK); vkw (BK): vehicle kerb weight; ES (BK): engine stress (estimated by the data extracted from the engine control unit); fc (BK): effective fuel consumption; ml (BK): maintenance level (information about ordinary and predictive maintenance performed by AI algorithm) [126].
1(CK)	$KPI_{fi}=f\left(KPI_{D_{i}},KPI_{V_{i}}\right)$	$KPI_{fi}=$ KPI of the fleet i including information of $KPI_{D_{i}}$ (CK) and $KPI_{V_{i}}$ that is KPI of the single vehicle $V_{i}$ (CK) (linear combination with specified weights as in Equation (1)).
1(CK)	$KPI_{E1}=f\left(lp,tr,\;tor,\ldots \right)$ As well as $KPI_{E2}=f\left(lp,tr,\;tor,\ldots \right)$	Indicator depending on the specific fleet. *lp*: load prediction for the specific region (BK); *tr*: traffic (BK); *tor*: type of road (BK) (highway, provincial road, mountain road, etc.).
1(CK)	$KPI_{E3}=f\left(fp,et,\ldots \right)$	Exogeneous indicators such as *fp* (BV): actual fuel price (BV); *et*: economic trend either of the fuels or of specific logistics services (BK).
2 (CK)	$KPI_{E\;level2}=f\left(\begin{array}{c} KPI_{fi},KPI_{E1},KPI_{E2},\\ KPI_{E3},M_{1},HR \end{array}\right)$	KPI combining information of *KP_fi_* (CK), *KPI_E_*_1_ (CK), *KPI _E_*_2_ (CK), *KPI_E_*_3_ (CK), *M*_1_ (CK) where *M*_1_ represents a process management indicator including logistics planning efficiency and vehicle management, *HR* (CK) indicates a human resource indicator about the correct choice of drivers (reliability, specific experience, etc.). *KPI _E level_*_2_ can be a “supernode” [104] reducing network complexity.
3 (CK)	$KPI_{E\;level3}=f\left(\begin{array}{c} KPI_{E\;level2}\left(fleet1\right),\\ KPI_{E\;level2}\left(fleet2\right) \end{array}\right)$	KPI “supernode” embedding information of *KPI_E level_*_2_ of the two considered fleets: this KPI represents the final “Energy” indicator of the whole complex system.

In Figure A4, an example of the KPI simulation related to the model of Figure A3 is shown. The proposed approach is useful to optimize logistics fluxes, taking into account an efficient energy behavior of the fleets. More complex systems can be associated with the joined actions of different vehicles involved in the transportation of the same product (transport by truck, or by plane, or by train, or by ship, etc.), and different logistics networks composed of different hubs.

## Appendix D. Example of Complex KPI in Photovoltaic Plants

Renewable energy systems are characterized by different elements such as renewable sources, local electrical networks (medium-voltage electrical cabling and electrical site components where the energy sources are allocated), and high-voltage networks. The complexity of the system is increased if different renewable energy fields are involved. In Figure A5, an example of a complex system associated with the monitoring and control of two PV fields transmitting energy to a high-voltage power plant is shown. The example refers to the model of two PV controlled fields structured in subfields. Their elements are:

- *Pi = f(Voltage, Current)*: PV dashboard reading generated voltage (BV) and generated current (BV);
- *Moti = f(SPVi)*: motor of the solar tracker controlling string orientation angle;
- *SPVi = f(**θ)*: solar sensor detecting maximum radiation (BV) due to the optimization of solar incidence θ);
- *WheatS = f(wind speed* (BV), *rain*(BV), *humidity*(BV), ...): sensors detecting weather parameters (wind speed, rain, etc.);
- *INVai = f(input current*(BV), *input voltage*(BV)): datalogger controlling inverter operation and input current and voltage (each inverter is installed in each subfield);
- *Trai = f(Converted Power)*: datalogger controlling transformer operation about the converted power from DC in AC (each transformer is installed in each subfield);
- *AI = f(SynPVi)*: artificial intelligence engine predicting malfunctions of each component of both the PV fields.

The KPIs of the analyzed system are listed in Table A2. All the variables are indicated in the graph of Figure A5.

**Table A2:** KPI related to each level of PV complex system to control (BV *is a basic value*, BK *is a basic KPI*, CK *is a complex KPI as a function of* BV *and* BK).

Level	KPI	Description
1(BK)	ST*k*a*t*PV*i* =*f*(*P_i_*, *Moti*); (*k* = 1,…*M*; *t* = 1,…*N*; *i* = 1,2,…*l*)	KPI as dashboard monitoring *M* number of PV strings related to *N* subfields and for *n* PV fields (monitoring *Pi* variables and solar tracker efficiency).
2(CK)	SynPV*i = f*(ST*k*a*t*PV*i, TRai*, *WheatS, INVai*); (*i* = 1,2,…*n*)	KPI as dashboard monitoring each PV field.
3(CK)	KPI_F_Tot = *f*(SynPV*i, AI*); (*i* = 1,2,…*n*)	Total indicator of the *n* PV fields.
4(CK)	KPI_Cab = *f*(KPI_F_Tot, *losses of high voltage cables*)	KPI indicator of 30 kV (nominal high voltage) cables connecting PV fields to the high-voltage power plant (monitoring of power losses as a function of the KPI_F_Tot (CK) and power losses of high-voltage cables (BV)).
5(CK)	KPITot = *f*(KPI_Cab)	KPI including all KPIs and high-voltage power plant components.
